# Integrins in the Immunity of Insects: A Review

**DOI:** 10.3389/fimmu.2022.906294

**Published:** 2022-06-09

**Authors:** Saima Kausar, Muhammad Nadeem Abbas, Isma Gul, Yu Liu, Bo-Ping Tang, Iram Maqsood, Qiu-Ning Liu, Li-Shang Dai

**Affiliations:** ^1^ State Key Laboratory of Silkworm Genome Biology, Southwest University, Chongqing, China; ^2^ School of Pharmaceutical Sciences, Wenzhou Medical University, Wenzhou, China; ^3^ Jiangsu Key Laboratory for Bioresources of Saline Soils, Jiangsu Synthetic Innovation Center for Coastal Bio-agriculture, Jiangsu Provincial Key Laboratory of Coastal Wetland Bioresources and Environmental Protection, School of Wetlands, Yancheng Teachers University, Yancheng, China; ^4^ Department of Zoology, Shaheed Benazir Bhutto Woman University, Peshawar, Pakistan; ^5^ Key Laboratory of Insect Developmental and Evolutionary Biology, Chinese Academy of Sciences (CAS) Center for Excellence in Molecular Plant Sciences, Shanghai Institute of Plant Physiology and Ecology, Chinese Academy of Sciences, Shanghai, China

**Keywords:** insects, hemocytes, integrins, innate immunity, target for pest control

## Abstract

Integrins are a large group of cell-surface proteins that are classified as transmembrane proteins. Integrins are classified into different types based on sequence variations, leading to structural and functional diversity. They are broadly distributed in animals and have a wide range of biological functions such as cell-to-cell communication, intracellular cytoskeleton organization, cellular signaling, immune responses, etc. Integrins are among the most abundant cell surface proteins in insects, exhibiting their indispensability in insect physiology. Because of their critical biological involvement in physiological processes, they appear to be a novel target for designing effective pest control strategies. In the current literature review, we first discuss the discovery and expression responses of integrins against various types of pathogens. Secondly, we examine the specific biological roles of integrins in controlling microbial pathogens, such as phagocytosis, encapsulation, nodulation, immune signaling, and so on. Finally, we describe the possible uses of integrins to control agricultural insect pests.

## Introduction

Integrins are a family of cell surface adhesion receptors that were first discovered in 1986 and have been found in all metazoans. Since their discovery, a tremendous amount of work has been done that substantially improved our understanding. They are non-covalently coupled heterodimers made up of two subunits (α and β), each of which is a single-pass type I transmembrane protein (1–3). The extracellular domains of integrins interact with proteins of the extracellular matrix in a unique way, making them highly versatile. In some cases, these receptor proteins bind to adjacent cells, promoting cell adhesion, which is important for providing mechanical support to a membrane, tissue maintenance and repair, embryonic development, hemostasis, and host defense. These physiological processes rely on short cytoplasmic tails of integrin proteins interacting with the intracellular cytoskeleton, which facilitates bi-directional force transmission across the cell membrane (4, 5). Integrins also convey chemical signals into the cell (outside-in signaling), providing information on the cell’s location, adhesive state, local environment, and surrounding matrix (2, 6). In addition to controlling cellular responses such as survival, differentiation, migration, and motility, it also serves as a context for responding to other signals such as those transmitted by growth-factor or G protein-coupled receptors. Integrins have the ability to modulate their affinity for extracellular ligands in addition to signaling from outside-in. In order to do this, they undergo conformational changes in their extracellular domains in response to signals that impinge upon the integrin cytoplasmic tails. This process is named as inside-out signaling or activation (7).

Integrin signals thus regulate a wide range of physiological processes in living organisms. Despite the plethora of information on the immunological responses of insects against different types of pathogens (8–10), no systematic literature review on the biological involvement of integrins in insect immunity has been published. In this review article, we discuss an overview of the biological role of integrins in insects’ immunity. In particular, we focus on the brief discovery of integrins in insects and describe the molecular mechanism by which integrins control various immune responses such as nodule formation, encapsulation, phagocytosis, melanization, immune signaling pathways, and others. In addition, we examine the interaction of integrins with other proteins or molecules for the accomplishment of immune functions.

## Discovery and Overall Structure of Integrins in Insects

Integrins are a large group of cell surface-adhesion receptors that are found in all metazoans. Integrins were discovered just over three decades ago and have since been extensively investigated in both invertebrates and vertebrates because they are indispensable for animal survival (11, 12). Integrins are transmembrane cell-surface glycoproteins of type I that are heterodimers made up of non-covalently linked α and β subunits (13). These subunits are constructed from different domains, with each subunit containing a large ectodomain that is important for ligand binding, a single transmembrane domain, and a cytoplasmic domain (cytoplasmic tail) that, in most cases, ranges in size from 20 to 70 amino acid residues (2, 14, 15).

Integrins have been reported in a variety of insect species, including *Bombyx mori, Manduca sexta*, and *Drosophila melanogaster*, etc. The number of integrins found in different species may vary. A recent study reported a total of eleven members, including α1-α3, αPS1- αPS3, and five *β* units (*β*1*- β*5) *B. mori* (16). While *D. melanogaster* possess Five *α* subunits (αPS1, αPS2, αPS3, αPS4, αPS5), and two *β* subunits (βPS and βν [beta-nu]) (17–20). Because *Drosophila* has tractable genetics, relatively much knowledge regarding how these integrin subunits are generated and how they play a biological role is available. The αPS1 subunit of *Drosophila*, which is encoded by the numerous edematous wings locus, is identical to the vertebrate subunits α3, α6, and α7, whereas αPS2, which is encoded by the inflated locus, is identical to α5, α8, αV, and αIIb (21, 22). In contrast, the remaining subunits (αPS3-αPS5) are not closely related to all of the vertebrate α subunits (18, 22). The αPS3 locus produces two transcripts and mutant alleles that induce phenotypic differences ranging from embryonic lethality with dorsal holes to adult viability (18). The putative ATG of αPS4 is only 259 bp downstream of the polyadenylation site of αPS3 transcripts, implying that it may depend on regulatory elements within the αPS3 gene and hence be part of the same complex gene. This could explain why apparent molecular null mutations in the αPS3 gen only cause a mild scab phenotype. It is unclear whether a strong scab affects both αPS3 and αPS4 subunits. In *Drosophila*, alternative splicing of the myospheroid transcripts encoding the βPS integrin subunit, a β integrin, leads to two distinct isoforms, each determined by the presence of one of two potential fourth exons (23, 24). The alternate exons, βps4A or βps4B, encode 29 amino acid residues in a region of the βPS subunit that has been involved in determining the ligand preferences of the human integrins. In the genome of *C. capitata* and other insect species, regions homologous to *Drosophila* βps4A and βps4B have also been reported (22). Furthermore, The expression of the second β subunit, *βv*, appears to be confined to gut endodermal cells (19). What α subunits form heterodimers with it is unknown. There are no known mutations in this gene, and embryo with a genetic defect in this region develops a morphologically normal gut (25).

Generally, animal genomes contain fewer β subunits compared to α subunits (26), suggesting that multiple α subunits can bind a single β subunit. For example, when one β subunit of *Drosophila* binds with three α subunits, three different functional integrin molecules are formed (20). The cytoplasmic domain of the β subunit is responsible for interacting with intracellular molecules that link integrins to the cytoskeleton and signaling molecules that transmit developmental signals (2). Thus, β subunits seem to play a crucial biological role in physiological processes, and their absence may result in functional abnormalities. The absence of β1 integrin subunits, for example, has been demonstrated to disrupt the organization of the mammalian embryo, mainly by inhibiting cell adhesion and spreading, resulting in cell death (27, 28).

## Integrins are Components of Immune Cells

Integrins are proteins that have been shown to be essential components of immune cells. Immune cells in vertebrates are termed as leukocytes. They are divided into T cells and B cells, dendritic cells (DCs), neutrophils, and monocytes, which differentiate into tissue macrophages (29). Leukocytes have been shown to be activated in response to immune signals inducing cells to migrate rapidly to infected sites in order to effectively respond to damage or infection (29, 30). Integrins are essential components of these cells that assist them in carrying out immune reactions (31). In contrast, in insects, Integrins show tissular specificity and appear to be an essential component of hemocytes. For example, In *P. includens* and *M. sexta*, α1 and α3 are mainly produced in hemocytes, α2 is widely transcribed in fat bodies but lowly expressed in hemocytes, and β1 is specifically expressed only in hemocytes (32–34). β1, on the other hand, is strongly expressed in fat bodies (35). While β1 is predominantly expressed in hemocytes in *O. furnacalis* (36). In *B. mori* α1 is highly expressed in hemocytes, α3 is also greatly expressed in hemocytes, α2 has a modest degree of expression in hemocytes, and β2 is expressed explicitly in hemocytes (37). These pieces of evidence indicate that, like vertebrates, plasma membranes of immunes cells in insects also contain high concentrations of integrins, implying that integrins are essential components of immunes cells in both vertebrates and invertebrates.

## The Biological Role of Integrins in Immunological Responses of Insects

**Figure 1:**
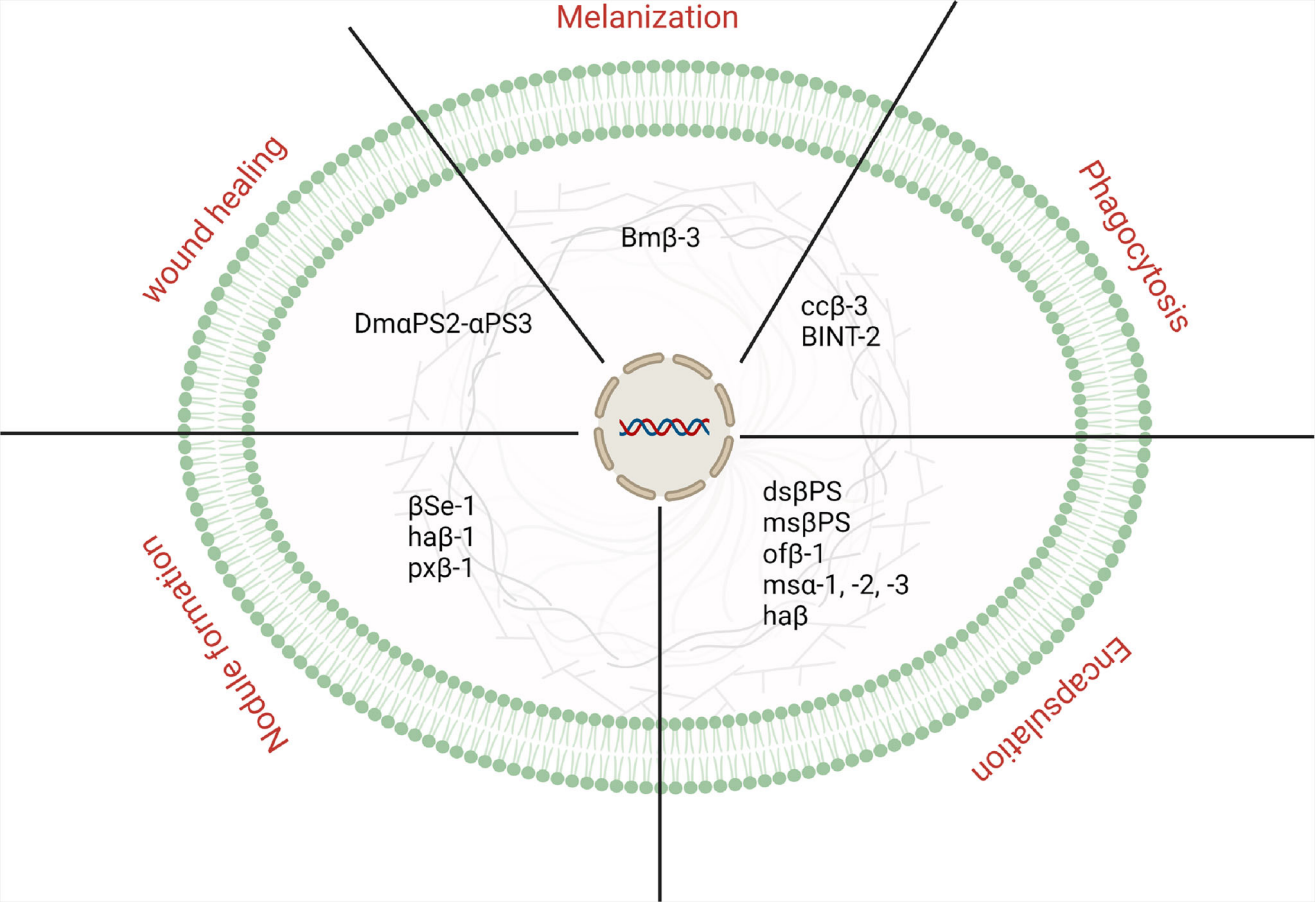
An overview is representing different physiological processes controlled by integrins in insects.

Besides the development and growth, which is not the focus of the present review article, integrins have been known to be involved in insects’ immune defense. Since their discovery, integrins have been identified and characterized in a number of insect species. In addition, growing evidence indicates that they play an effective biological in the defense of insects. This section discusses the immunological importance of integrin (**Figure 1**).

### Integrins in Biotic Stress Responses

Growing evidence suggests that integrins production is increased in response to infection. In *B. mori*, β3 integrin has been shown to be extensively produced in response to bacterial and pathogen-associated molecular patterns (PAMPs), implying that these proteins are likely involved in hemocyte-mediated immunity (37). Another study recently observed comparable expression patterns of integrin proteins in hemocytes, confirming the hemocyte-associated biological roles of integrins (35).

Therefore, it was considered that integrins are only associated with hemocytes or blood cells in animals (29, 37). In contrast, a recent study on *B. mori* suggested that integrins are found in hemocytes and also expressed in other tissues, including fat bodies (35). The fat body is a multifunctional tissue that involves integrating signals, synthesizing immune system proteins, serving as a center of metabolism, regulating molting and metamorphosis, and synthesizing hormones that regulate body functions. Apart from their traditional roles in hemocytes-mediated immunity, integrin may also play a role in influencing the synthesis of immune proteins and hormones, hence regulating pathogen infection in insects. However, evidence is required to confirm their specific immune roles in the fat bodies (38). Zhang and his co-workers (2017) suggested that β3 integrin increases the expression of IMD, and Toll pathway-related genes in hemocytes, suggesting that likely these proteins may control the production of immune-associated factors. However, the authors failed to elaborate on the molecular mechanism by which integrin influences immune signaling pathways. In addition, *Spodoptera exigua* remarkably upregulates the expression of βSe1 during Gram-negative bacterial or fungal infection, and it shows a similar expression pattern after lipopolysaccharide or laminarin challenge; however, Gram-positive bacterial infection had no effect on expression levels (39). Another study used a different approach, challenged the *Helicoverpa armigera* with beads, and analyzed expression patterns of integrin α-PS1 at different time points. This study suggested that α-PS1 is highly induced after the bead challenge in hemocytes (40).

So far, only a few studies are available that directly discuss the responses of integrins against microbial pathogens. *In vivo* expression analysis indicates that integrins production is increased in the presence of various pathogens, such as Gram-positive bacteria, Gram-negative bacteria, or beads. The upregulation of these proteins is clear evidence that they have crucial biological roles in insect immune responses. Although the studies into the biological involvement of integrins in stress response are relatively restricted, these studies suggest that different integrins play a role in response to diverse stress conditions in insects.

### Integrins in Phagocytosis

Phagocytosis is an evolutionarily conserved process in which hemocytes recognize, internalize and eventually eliminate apoptotic cells and invading microbial pathogens. In the hemolymph of insects, granulocytes or plasmatocytes are the only hemocytes responsible for phagocytosis (41–43). Various molecules and receptors residing on the surface of membranes, such as integrins, are involved in the binding and internalization of microbial pathogens.

**Figure 2:**
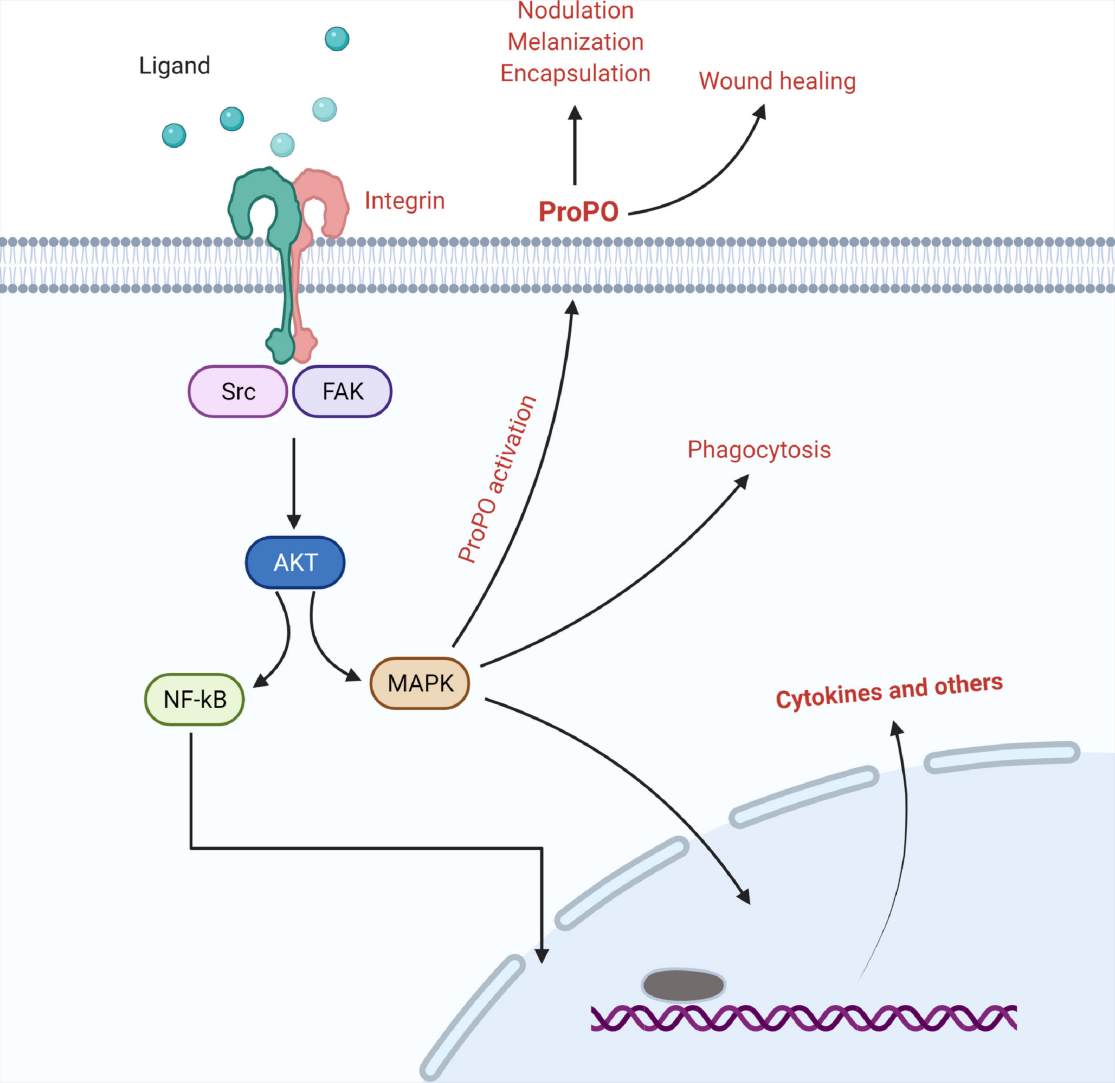
The involvement of integrins in the regulation of nodulation, melanization, encapsulation, and wound healing *via* focal adhesion kinase and its associated signaling.

Integrins are essential surface receptors that mediate signal transduction between the extracellular matrix and the cytoskeleton, influencing morphological changes in hemocytes, which are usually attributed to cytoskeletal rearrangements that occur during phagosome formation (**Figure 2**) (15, 44). Many members of the integrin family have been shown to have a role in the process of phagocytosis; however, their mechanisms of action may vary depending on the type of molecule. For example, in the Mediterranean fruit fly, *Ceratitis copitata*, integrin β3 serves as a receptor and stimulates cytoskeletal rearrangement for *E. coli* phagocytosis, and their binding activates integrin specific signal of pathogen internalization (45, 46). For the engulfment of *E. coli*, the β integrin subunit in the surface of medfly hemocytes transmits signals to focal adhesion kinase (FAK) and its downstream targets, including Src, Elk-1-like protein, and MAP kinases. The family of MAP kinase is an established intracellular evolutionary conserved phosphorylation cascade that regulates inflammatory responses, apoptosis, and phagocytosis (47, 48). In addition, these investigations suggested that integrins can only bind to gram-negative bacteria and cannot interact with gram-positive bacteria (45, 49).

Phagocytosis has also been demonstrated in *Anopheles gambiae* and *Ceratitis capitata* (medfly), where the β subunit has been found to regulate the bacterial phagocytosis (49, 50). In *Anopheles gambiae* immune-responsive cell line, RGD ligand recognition receptors play a critical biological role in the phagocytic response, showing the overlap between molecular components participating in adhesion and phagocytosis. BINT2, a newly discovered integrin family member, has been shown to play a vital function in the phagocytosis of *E. coli* by a hemocyte-like cell line. Furthermore, *in vivo* suppression of BINT2 in mosquitoes (*A. gambiae*) results in a remarkable reduction (more than 70%) of *E. coli* phagocytosis (50, 51). It has been hypothesized that BINT2 protein can either directly bind to bacteria or indirectly recognize bacteria: BINT2 protein binds to an RGD containing lectin, which recognizes *E. coli* (50). Another study demonstrated an approximately similar phenomenon, in which this study identified globule-EGF-factor 8 (MFG-E8), which is produced by thioglycollate-elicited macrophages that links apoptotic cells to phagocytes. MFG-E8 specifically interacts with apoptotic cells by recognizing aminophospholipids such as phosphatidylserine. MFG-E8, when engaged by phospholipids, binds to cells *via* its RGD (arginine-glycine-aspartate) motif—it binds particularly strongly to cells expressing α_v_β_3_ integrin. The NIH3T3 cell transformants that expressed a high level of α_v_β_3_ integrin have been found to engulf apoptotic cells when MFG-E8 is added (52). Therefore, further research into the binding mechanism of this newly identified protein (BINT2) is needed.

In contrast, many recent studies argue that integrin β can bind to pathogen-associated molecular patterns (PAMPs) derived from Gram-positive and Gram-negative bacteria, such as LPS and PGN, implying that they are responsive to a wider spectrum of bacterial pathogens (37, 53–55). reported the ability of *B. mori* β3 integrin to bind with LPS *in vivo*. The interaction of β3 integrin with PAMPs, suggests that after binding, these proteins induce signals that cause the infectious particles to internalize, which is then responsible for phagocytosis. In comparison to PAMPs, the authors did not detect any interaction between recombinant β3 integrin protein and *E. coli* or *P. aeruginosa* bacteria *in vitro*. The possible reason for this weak interaction between them could be attributed to the weak binding of the recombinant protein to *E. coli* or *P. aeruginosa.* In addition, the other possible reason is that the recombinant integrin protein used in this study is likely not folded precisely *in vitro* (37). However, this phenomenon is of great interest to determine what biochemical changes occur in the structure of integrin β3 when it is expressed in the prokaryotic expression system, as these changes could limit the ability of the recombinant protein to bind to bacterial pathogens *in vitro*. Furthermore, these contrasting results suggest that integrin protein binding ability may vary in different species or some unknown mechanism controls their binding.

However, a recent study in the same species *B. mori* suggested that integrin β1 can interact with Gram-positive bacteria, S. aureus in the presence of CaCl2 in addition to Gram-negative bacteria, including *E. coli*, or *P. aeruginosa* (35). In another insect species (49), knockdown of βps4A and βps4B in medfly hemocytes reduce hemocytes capability by about 40% to uptake bacteria. In addition, the authors incubated hemocytes with gram-negative (*E. coli*) or gram-positive (*S. aureus*) bacteria in the presence of anti β1 and β3 human antibodies; subsequently, they found an almost 20% reduction in the uptake of bacteria by hemocytes.

Integrins have wide-spectrum activity against Gram-negative and Gram-positive bacteria, as evidenced by the examples above. The binding and elimination of bacterial pathogens by different types of integrins may differ. This ability may differ between species, implying that integrins may serve species-specific functions. Future studies on different insect species may help to clarify these inferences.

### Integrins in Encapsulation

Encapsulation is the primary response of lepidopteran hemocytes against microbial pathogens, and hemocytes bind to non-self-molecules (e.g., microbial invaders) during this process. Hemocytes create a multilayered cellular sheath after attaching to microbial invaders, which is followed by melanization (42, 56). There are multiple lines of evidence that integrins are involved in insects’ cellular immune response by encapsulation (**Figure 2**) (32). The ability of lamellocytes to encapsulate has been shown to be disrupted in a βPS integrin loss of function mutant in *D. melanogaster* (57). Another study found that when integrins are suppressed by specific RNA interference, hemocyte-mediated encapsulation against microbial invaders in *M. sexta* is impaired (33, 34, 58). Integrin β1 from *Ostrinia furnacalis* has also been shown to influence the encapsulation process of plasmatocytes (36, 44). Additionally, although encapsulation and melanization are two distinct processes, melanization usually occurs together with encapsulation to remove foreign invaders (59). In *Drosophila* hemocytes, PS integrin is required for encapsulation reaction. Following encapsulation, *Drosophila* hemocytes induce the prophenoloxidase gene, which generates the enzymatic proteins essential for melanization of the capsule formed during encapsulation (57).

In *Manduca sexta*, α1, α2, and α3 integrins have also been reported to facilitate encapsulation, in particular α1 and α2. The α1 hemocyte-specific integrin contains a β subunit that has the capability to interact with the LEL domain of tetraspanin D76, suggesting that tetraspanin (Integral membrane proteins family with four transmembrane domains) on one cell surface interacts with hemocyte-specific integrin on another cell surface. This integrin-tetraspanin association is unique in insects and has not been reported in mammals (33, 60). In contrast, the α2 subunit is related to integrins with RGD-binding motifs and thus binds to substrates. However, all three α-integrin subunits are expressed on hemocytes during the immune response, and exposure of hemocytes *in situ* to dsRNAs that disrupt the expression of each of these α subunits also abolishes encapsulation (34). Overall, the individual *α* integrin subunits of *M. sexta*, like their mammalian immune system counterparts, have crucial, individual biological roles in cell-cell and cell-substrate interactions throughout cellular immune responses. Furthermore, activation of ligand-binding by the hemocyte-specific β1 integrin plays a critical biological role in inducing plasmatocyte adhesion leading to encapsulation. *In vitro* studies revealed that recombinant protein containing the I-like domain can bind to MS13 and MS34 (mAbs specific for *M. sexta* hemocytes), blocking their ligand-binding site and subsequently impairing the adhesion of plasmatocytes. In contrast, inhibiting integrin *β*1 in plasmatocytes resulted in a remarkable reduction in encapsulation (33).

Integrin has been proposed to serve as a C-type lectin receptor (CTL3) during the encapsulation reaction of hemocytes. This physical interaction between pattern recognition receptors and integrins promotes encapsulation reaction in insects, as it modulates the physiological modifications in cells throughout the encapsulation process (61). A recent study using *in vitro* and *in vivo* analysis demonstrated that CTL3 promotes encapsulation and melanization reactions in *Helicoverpa armigera*, while β integrin contributes to the encapsulation reaction. Co-immunoprecipitation analysis of CTL3 interacts with β-integrin, suggesting their strong interaction that simultaneously improves the encapsulation reaction. Further, this study observed that suppression of β integrin could reduce encapsulation reaction in CTL3-coated beads. Interestingly, the authors observed that 20-hydroxyecdysone (20E) administration could enhance CTL3 transcription and inferred that the interaction of pattern recognition receptors and integrin is governed by steroid hormone ecdysone and thereby it regulates the encapsulation process (62).

### Integrins in Nodule Formation

Nodulation is a biphasic cellular immune response in insects that begins with hemocytes secreting adhesion molecules, followed by granulocytes entrapping microorganisms in clusters and forming aggregates with other granulocytes. Plasmatocytes eventually wall off these microaggregates, forming a nodule (63, 64). The attachment of hemocytes is mediated through cell surface adhesion molecules during nodulation and encapsulation. Hemocytes of lepidopteran insects contain cell surface integrin proteins that are involved in the adhesion of cells (34, 37, 65). Integrin proteins on plasmatocytes in *M. sexta* are derived in part *via* interactions with neuroglia and a tetraspanin on neighboring hemocytes (58).

Several studies have suggested that integrins play a biological role in immune responses by forming nodules in insects (**Figure 2**) (39, 66, 67). Surakasi et al., found that βSe1 stimulates the formation of nodules in *S. exigua*, which aids in the removal of pathogens (39). The authors also demonstrated that βSe1 suppression significantly decreases hemocyte-spreading behavior and nodule formation against bacterial challenges (39). Similar evidence has also been shown in *P. xylostella* and *Helicoverpa assulta*, where a deficiency of β1 reduced nodule formation following bacterial challenge (66, 67). Contacts with extracellular matrix or hemocyte-hemocyte interactions can cause hemocyte behavior to be aggregated. *M. sexta* secretes neuroglian, a cell adhesion molecule, on the cell surfaces of granular cells and a subset of large plasmatocytes (58). Hemocytes that are positive for neuroglian act as aggregation foci against foreign surfaces (68). Because some mammalian integrins can bind to at least three different members of the immunoglobulin superfamily: ICAM-1, ICXAM-2, and ICAM-3, the Immunoglobulin domain of neuroglian has been suggested to be a ligand of integrin system (69). Thus, it seems that β1 subunit is required to carry out the processes of hemocyte cellular immune responses. In insects, Cell-mediated responses of the immune system involve hemocytes quick transformation from their resting non-adherent states to their active adherent states (70). Hemocytes then form attachments with one another, allowing cell-cell interactions to form nodules (32). Foreign substrate recognition following immune challenge can induce β1 conformational change to transform hemocyte integrins into an active adhesive state to be involved in various cellular immune responses of *S. exigua*, based on a coupling role of integrins between the cell membrane and prediction of putative phosphorylation sites in βSe1 (39). Overall, the pieces of evidence indicate that integrins are an important component of plasma membranes that indirectly trigger adhesiveness in plasma membranes and ultimately cause nodule formation.

### Integrins Modulate the Melanization Process

The Toll and IMD pathways drive the expression of antimicrobial peptides (AMPs) and other immune responsive genes in insects and hence play a crucial biological role in the innate immune system (8, 9, 71). However, these immune pathways take a few hours to a few days to induce their immune effectors. On the other hand, melanization is a more immediate immune response that occurs just a few minutes after infection. Melanization involves the oxidation of phenols to quinones, which then polymerize to produce melanin. Melanin is deposited around them to help sequester infiltrate microbial pathogens at wound sites. Microbial pathogens are thought to be directly harmful to the quinone substances and other reactive oxygen intermediates generated during melanization (8, 72). In addition, other processes in immune responses such as blood coagulation, wound healing, phagocytosis, and AMPs expression have been found to work together with the melanization reaction (8).

Integrins have been implicated in melanization processes in some invertebrate species. For example, A recent study on *Litopenaeus vannamei* has suggested that integrin β plays an important role in proPO activation (73). However, in insects, only one study reported a relationship between integrin and melanization (**Figure 2**) (37). The authors suppressed integrin β3 in the larval stage of a lepidopteran model species, the silkworm, *B. mori*, and then challenged with bacterial pathogen (37). After reducing integrin β3, the authors reported that hemolymph melanization increased. They also reported an increase in the transcription of melanization-related genes (PPO1, PPO2, BAEE, SPH78, SPH125, and SPH127). Interestingly, these findings are in contrast with the previous study of Lin et al. on crustaceans, who reported a decrease in the PO activity after β integrin knockdown (73). So far, the molecular mechanism by which integrins participate to control negatively or positively has not been discovered in crustaceans and insects. In addition, it appears that proPO activation occurs in the presence of PAMPs, which are recognized and bound by PRPs like LGBP, and the PAMP–PRP complex reacts with integrin (74, 75), suggesting indispensability of integrins for the PO activation. However, this mechanism may vary in different groups of animals or may species specific. Future studies may highlight how integrin-govern melanization process.

### Integrins Regulate Immune Pathways

**Figure 3:**
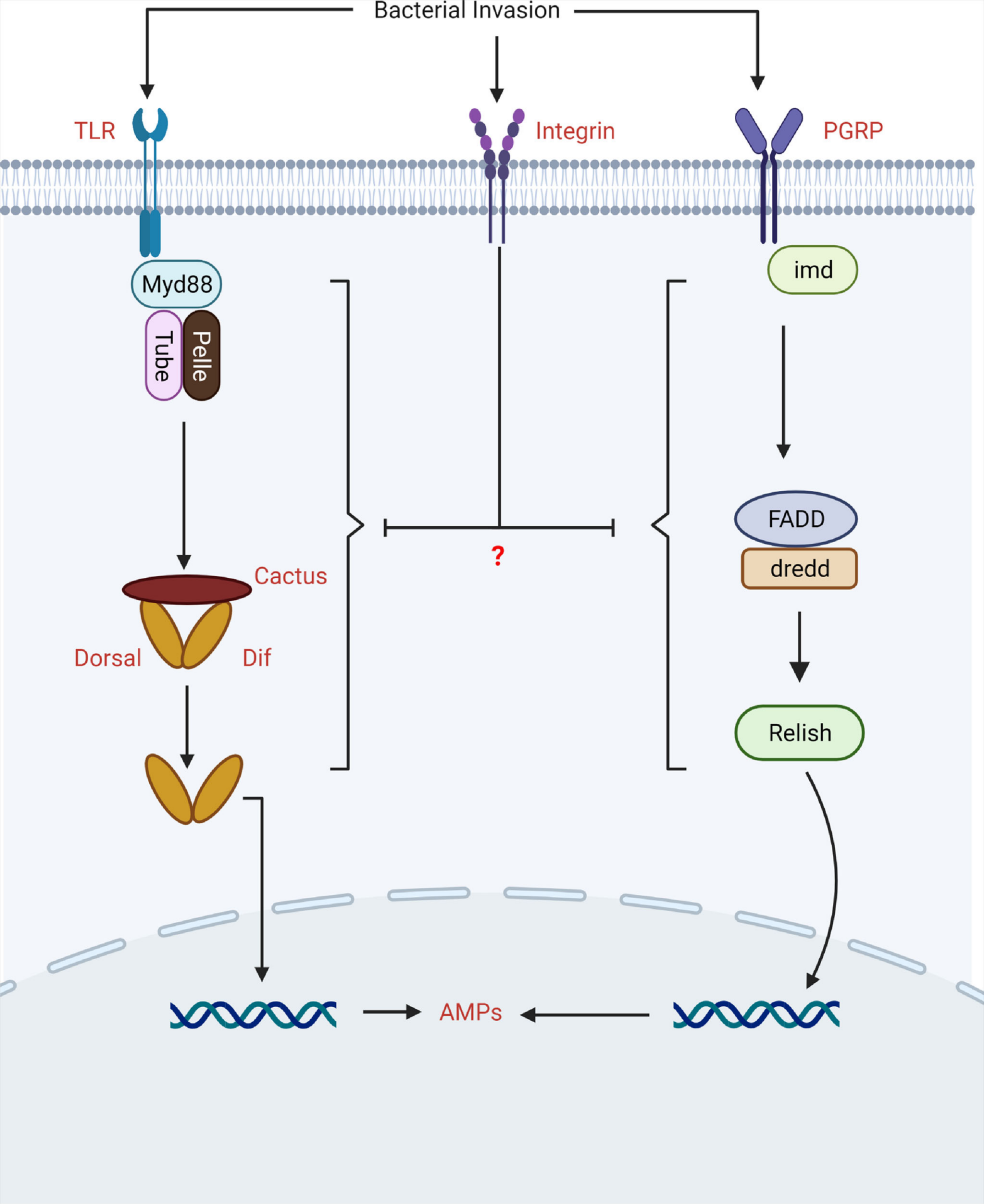
The biological role of integrins in the modulation of Toll and IMD signaling.

Toll-like receptors (TLRs) and a growing number of non-TLR receptors are responsible for the detection of pathogens. For example, integrins have also been shown to act as pattern recognition receptors and modulate downstream signaling (e.g., Toll and IMD) in order to stimulate immune factors (**Figure 3**) (76). The first evidence in insects came from the study of Zhang and his colleagues, who discovered that RNAi knockdown integrin influence the innate immune responses of *B. mori* by affecting immune signaling. The authors noted that silencing of β3 in the presence of bacterial challenge induced Toll and IMD signaling. They found that loss of β3 integrin increased the expression of Toll (Relish and FADD), and IMD (TRAF2, Pelle, and Tube) pathway-associated genes, implying that integrin β3 could negatively modulate Toll and IMD signaling pathways in insect. However, the authors did not study the mechanism behind the upregulation of Toll and IMD pathway-associated genes in the absence of β3 integrin (37).

In contrast, in vertebrates, Gianni and his co-workers suggested that αvβ3 integrin is an important sensor and activator of specific components of the innate responses to herpes simplex virus-1. By loss- and gain-of-function assays, αvβ3 integrin has been shown to be crucial for the production of type 1-IFNs and of a specific set of cytokines, which is a major determinant in NF-κB activation and reduces viral growth in a single replication cycle. Mechanistically, the αvβ3 integrin relocates the herpes simplex virus-1 receptor nectin1, thus herpes simplex virus-1, to cholesterol-rich membrane microdomains, where the virus is endocytosed while also triggering the innate immune response (77, 78). For herpes simplex virus-1 and possibly for a number of other viral and bacterial pathogens, αvβ3 integrin seems to be a non-TLR pattern recognition receptor (76). In the case of HSV infection in vertebrates, αvβ3 detects and binds to virion glycoprotein, which in turn binds to TLR2 (79).

Collectively, only one study in insects identified the integrin as a pattern recognition receptor that can negatively regulate the expression of Toll and IMD pathway-associated genes, which seems to be in contrast with vertebrates. Future studies need to evaluate how integrins work differently in different taxons. If they do so, then how do insect integrins negatively modulate immune signaling pathways. More research on diverse insect species may further highlight how integrins govern Toll and IMD signaling pathway pathways. In addition, these may also analyze the molecular mechanisms by which integrins modulate these immune signaling pathways.

### Integrins Involvement in Wound Healing

A skin wound exposes underlying tissues and the entire organism to further damage and infection, so it must be healed quickly. “Reep-ithelialization” (RE) is a vital step of wound healing in which sheets of skin cells migrate toward and reseal the wound. Our knowledge of reep-ithelialization mechanisms is informed by studies of single migratory cells that must polarize, generate functionally different front and rear sides, as well as engage contractile mechanisms to exert force. Finally, controlled adhesion and deadhesion enable movement across a substratum, typically the extracellular matrix. Small GTPases of the Rho family mediate front/rear polarization (80–82), and actomyosin supplies propulsive forces, with actin polymerization driving lamellipodia extension (82, 83). Integrins are the major receptors for the extracellular matrix and are essential for cell crawling (84, 85). Extensive but poorly understood mutual regulation among the Rho-GTPases, actomyosin, and integrins directs the forward migration of cells (80, 82, 86).

Integrins, heterodimers of α and β transmembrane glycoproteins that bind the extracellular matrix, are especially important in reep-ithelialization. Integrins nucleate large cytoplasmic complexes that not only tune adhesion in response to both intracellular and extracellular cues (87, 88) but also engage in bidirectional signaling, affecting cytoskeletal activities and gene expression inside the cell (89) and shaping extracellular matrix composition on the outside (90, 91). Integrin abundance and function are also regulated by transcriptional modulation (92), switching of different integrins (93, 94), protein clustering and localization (95), vesicle trafficking (96), and protein turnover (97, 98).

A recent study using *in vivo* analysis reported the involvement of αPS2-βPS and αPS3-βPS as the crucial integrin dimers and talin as the only integrin adhesion in the reepithelialization (99). The authors noted severe reepithelialization defects in βPS, PS, and talin (integrin adhesion component) deficient *Drosophila* larvae, notably in αPS2-αPS3 pair depletion, indicating that PS2 and PS3 integrins play a crucial role in larval epidermal wound closure. The expression of these proteins is enormously increased in the wound surrounding cells in a JNK-dependent manner (99, 100). After that, the integrins rapidly accumulate in a few rows of cells surrounding a wound. Intriguingly, the integrins localize to the distal margin in these cells instead of the frontal or lamellipodial distribution expected for proteins providing traction and recruiting non-muscle myosin II to the same location (99).

Another study analyzed the process of polyploidization, which is essential for wound healing. Polyploid cells appear in adult tissues, at least in part, to promote tissue repair and restore tissue mass. However, the signaling required for polyploid cells in response to injury in insects has recently been discovered by Besen-McNally et al. (101). The authors demonstrated that wound-induced polyploid cells are generated by cell fusion and endoreplication, resulting in a giant polyploid syncytium in the adult *Drosophila* epithelium. They further showed that the integrin focal adhesion complex is an activator of wound-induced polyploidization. Both integrin and focal adhesion kinase are upregulated in the wound-induced polyploid cells and are required for Yorkie-induced endoreplication and cell fusion, as evidenced by wound healing is perturbed when focal adhesion genes (Mys and Fak) are knocked down (101). However, the focal adhesion complex that regulates cell fusion and formation of syncytium other signaling mechanisms may also derive wound closure in insects that may also be connected with integrin proteins that are still unidentified.

## Integrins as a Specific Target for Pest Control

Various insect groups, such as coleopterans, lepidopterans, and aphids, have a number of well-known pest species, the majority of which are voracious feeders of plant materials (102–104). So far, a variety of chemical and biological control strategies have been applied to keep their population well below a threshold level. On the other hand, insect pests have developed sophisticated defense tactics to adapt to various insecticidal materials (105). This scenario demands the development of a new targets arena and their effective regulators.

Integrins have been demonstrated to have a role in a variety of physiological processes in insects, making them suitable pest control targets. Integrin receptors mainly modulate immune responses, development, growth, molecular signaling, and others (35); for example, Mohamed et al. suggested that integrin may be used as a specific target to control the lepidopteran pest, *P. xylostella* (66). The authors noted that the suppression of βPx1 by double-stranded RNA has an impact on the developmental and immune activities of this species. They showed that larvae treated with dsβPx1 had a slower developing rate and that those that survived metamorphosed into relatively small pupae. This could be due to a lack of βPx1, which could cause multiple disruptions in different physiological processes in which integrins are involved in cell-cell interactions. In addition, silencing of integrin β1 increases the mortality of *Plutella xylostella*. This further showed that dsRNA has oral toxicity in young immature stages, with toxicity based on RNA interference (RNAi) being more specific to *P. xylostella* in a dose-dependent manner. When a transgenic plant producing dsRNA is used to manage insect pests in the field, this specificity is extremely important because transgenic plants can be exposed to a variety of plant herbivores. The oral toxicity of dsβPx1 implies a novel transgenic-plant-based strategy for controlling *P. xylostella* (66).

**Figure 4:**
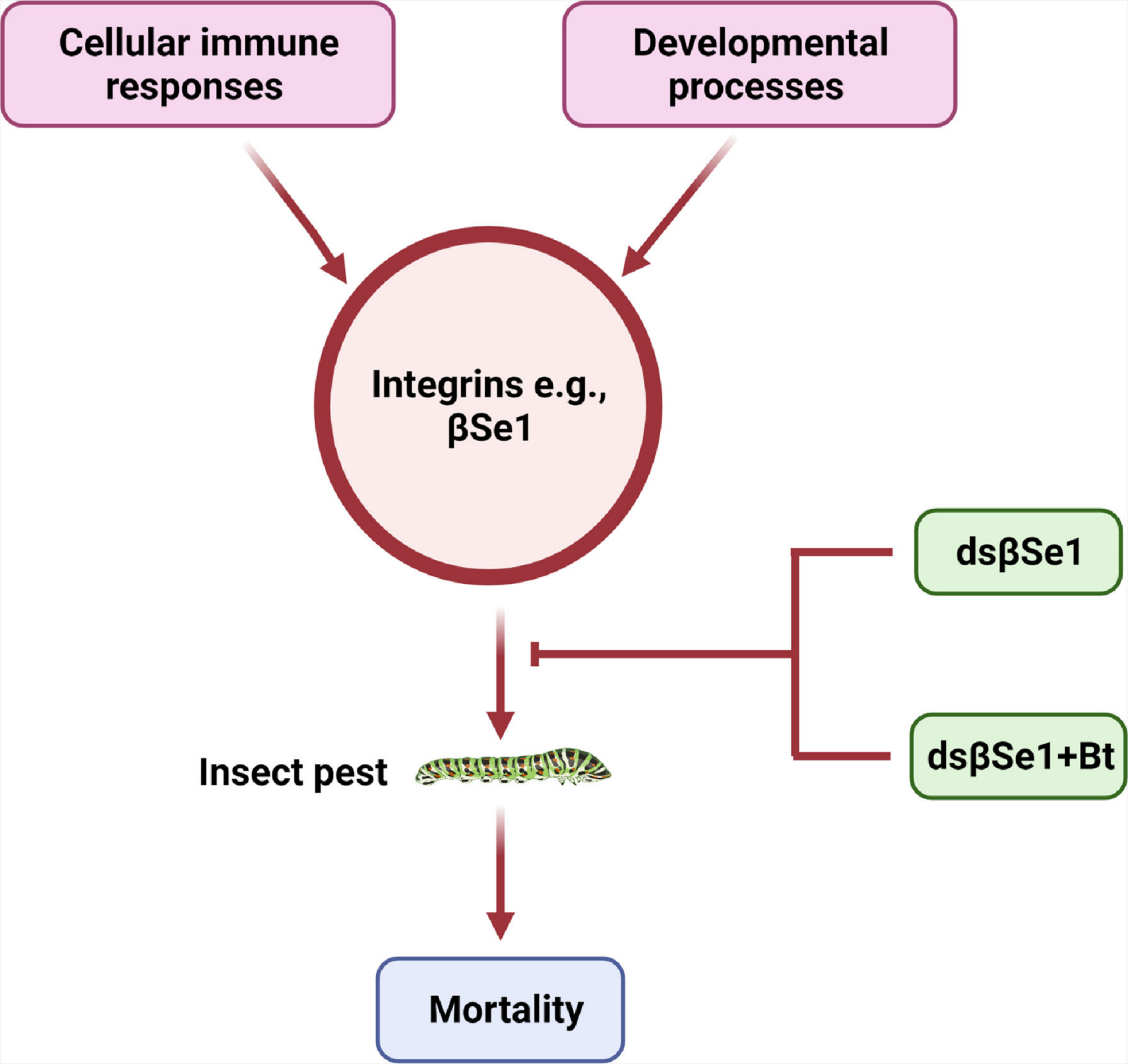
Integrins as a target for insect pest control, e.g., integrin βSe1 is vital for the development, cellular immune responses, and the survival of insects. A novel class of insecticidal compounds can be generated by using one more of the potential strategies.

*Spodoptera exigua* is a polyphagous lepidopteran pest with a broad distribution that causes severe economic damage to agricultural crops (18). βSe1 has been shown as an important target for controlling *S. exigua* (39). Surakasi et al., synthesized double-stranded RNA against βSe1 (dsRNA^βSe1^) and administered it to larvae orally. The pupal weight was substantially reduced as a result of oral feeding. The dsβSe1 treatment also impaired innate immune responses of *S. exigua* in response to bacterial challenge. Furthermore, oral ingestion of dsβSe1 triggered reduction of βSe1 expression in the midgut and resulted in considerable mortality of *S. exigua* during immature development, implying that βSe1 could be an effective target for controlling this pest species (39). Another study further highlighted the use of βSe1 as a pest management target site (106). This study provides an efficient approach for using dsRNA specific to an integrin gene by mixing it with a biopesticide, *Bacillus thuringiensis* (Bt). Similar to Surakasi et al., this study noted that transformed *E*. *coli* expressing dsβSe1give has a strong oral insecticidal effpicacy against young larval instars and delays the larval development. Some of the larvae have a miniature body form, which was most likely due to malnutrition induced by dsβSe1 damage to the midgut (39, 106). Kim et al., further suggested that the transformed bacteria expressed the dsRNA, and the amount of dsRNA produced was proportional to the number of bacterial cells. It indicates that one recombinant *E. coli* can produce 2.8 ± 0.1 pg of dsRNA as the authors demonstrated based on the total dsRNA amount and the bacterial cell counts. In addition, the authors demonstrated that 350 ng of dsRNA to be effective to give a maximal insecticidal activity by feeding the transformed *E*. *coli* because they found the maximal mortality from the bacterial treatment at 10^7^ cells per larva. This insecticidal activity of dsβSe1could be triggered by a transformed bacterial treatment producing dsβSe1 that induces a serious impairment of the midgut epithelium. Finally, the efficacy of Bt toxin in combination with bacteria expressing dsβSe1. For this purpose, Kim et al., suggested that for the effective combinational activity of the bacteria expressing dsβSe1 to Bt toxin, βSe1 should be suppressed before the Bt treatment because the Bt toxicity increased with the elapse time after feeding the dsINT-expressing bacteria (106). A similar bacterial treatment study in *S*. *exigua* showed that it took more than seven days after the feeding (107). However, Kim et al. found that the effective RNAi effect appeared at 3 days after the bacterial feeding treatment to *S*. *exigua* larvae, at which the Bt efficacy was significantly enhanced (106). Thus, this increased effeicacy combinational treatment is an important technique to enhance the insecticidal efficacy of current Bt crops by supplementation with expression of dsRNA specific to integrin (**Figure 4**).

The evidence provided above was sufficient to use βSe1 as a target sit for controlling *S. exigua* in the laboratory conditions. Later, Kim and Kim, developed a formulation technique of the dsRNA-expressing bacteria for applying the bacterial insecticide field populations (108). The authors formulated the recombinant bacteria by freeze-drying method and then tested its control efficacy against target insects. They found that the formulation had substantial insecticidal activity against the last instar larvae of *S. exigua*. In contrast, commercial Bt insecticide exhibits only about 60% insecticidal activity against *S. exigua* last instar. However, the authors did confirm that combining this formulation remarkably increased Bt insecticidal activity. They also suggested that integrin-expressing bacterial formulation exhibits relative selectivity to target insects depending on sequence similarity (108). These results suggest that βSe1 could be a key target to control pest species; however, insecticide products of this integrin seem to be species-specific or may be useful in species with integrin that have high similarity to βSe1.

*Helicoverpa assulta*, is an oligophagous insect that feeds on a wide range of commercially important plants (109). In *H. assulta*, the integrin β1 subunit is strongly produced in the gut and hemocytes, suggesting that it could be a promising target for insecticide development. Park et al. suggested that dsβ1 treatment increase *H. assulta* larval more susceptible to pathogenic Bt Cry toxins and *X. nematophila* (67). Thus, it seems that RNAi treatment of β1 integrin subunit in *H. assulta* may weaken the interaction of the resistant factor with Bt receptors and may enhance toxicity. These suggest that using dsRNA specific to the β1 subunit of integrin is a novel control strategy for *H. assulta*. Furthermore, the effects of RNAi on larval susceptibility to pathogens indicate that the combination of dsRNA and pathogenic bacteria to synergize the microbial pesticide control efficacy (67).

Overall, the researchers suggests that integrins could be a key target for developing effective insecticides against insect pests in the future. Although many integrins from different species have been shown to be target candidates for insecticides development, only the βSe1 integrin has been reported to be used as a formulation and its effectiveness (108). Therefore, future studies should focus on identifying more promising integrins candidates for insecticide synthesis in various insect species. The identified candidate then needs to use specific formulations under field conditions as well as evaluate their efficacy to cross-species.

## Conclusion and Future Aspects

Integrins have been a topic of interest for several years due to their importance as a component of the cell membranes and their involvement in the development and the immune system. Insects and other invertebrates integrins seem to be structurally and functionally similar to vertebrate integrins, as indicated by significant progress in a functional study on insect integrins. Although, significant progress has been made on the biological roles of insect integrins over the last decades, but there is no comprehensive review so far on integrins regarding the immune system of insects. Integrins have been described in a variety of insect species, but their biological roles and molecular mechanisms are still unknown for most of the integrins. Additional studies on identification and functional characterization in insects are likely to be resolved remaining questions about the structural features and molecular mechanisms of integrin. With the complete sequencing of genomes, we will have access to the full complement of integrin subunits in organisms. Also, the further advancement of molecular genetic methods employed in the context of cell biology questions will permit a wider range of functional studies. Because integrin structure and function in invertebrates are similar to that of vertebrates, these studies will remain relevant and potentially may contribute substantially to our understanding of this remarkable group of receptors. Integrin negatively regulates the production of antimicrobial peptides; however, the mechanisms by which it mediates signaling are undiscovered. For example, beta integrin 3 of *B. mori* has been shown to control antimicrobial peptides, but how it controls this process is still unknown. In addition, to further ensure the regulatory role of the immune signaling cascade, a comparative study is needed. Our understanding of the molecular basis of these processes continues to advance, bringing with it the potential for strategies that modulate integrins and their associated signaling for the management of commercial insects and control of agricultural pests.

## Author Contributions

The authors’ responsibilities were as follows: L-SD, Q-NL and IM designed this review article. L-SD, SK, MNA, IG, YL, and B-PT downloaded material and write down draft. L-SD, Q-NL and IM draw the digram and proofread the article. All authors contributed to the article and approved the submitted version.

## Funding

This work was funded by the Natural Science Foundation of Zhejiang Province, China (No. LQ20C190009), the Natural Science Research General Program of Jiangsu Provincial Higher Education Institutions (21KJA240003), the Natural Science Foundation of Jiangsu Province (BE2020673), the Doctorial Start-up Fund of Southwest University (SWU020023), the National Key R&D Program of China (2019YFD0900404-05), the Industry-University-Research Cooperation Project of Jiangsu Province (BY2020644), the Open Funding Project of Anhui Province Key Laboratory of Aquaculture & Stock Enhancement (AHSC202001), the 16th Six Talents Peak Project of Jiangsu Province (NY-126), the National Natural Science Foundation of China (32070526), the Jiangsu Agriculture Science and Technology Innovation Fund (CX(18)3027). The study was sponsored by the Qing Lan Project of Jiangsu Province, and the ‘Outstanding Young Talents’ of YCTU.

## Conflict of Interest

The authors declare that the research was conducted in the absence of any commercial or financial relationships that could be construed as a potential conflict of interest.

## Publisher’s Note

All claims expressed in this article are solely those of the authors and do not necessarily represent those of their affiliated organizations, or those of the publisher, the editors and the reviewers. Any product that may be evaluated in this article, or claim that may be made by its manufacturer, is not guaranteed or endorsed by the publisher.
